# Tactile Spatial Acuity in Childhood: Effects of Age and Fingertip Size

**DOI:** 10.1371/journal.pone.0084650

**Published:** 2013-12-19

**Authors:** Ryan M. Peters, Daniel Goldreich

**Affiliations:** Department of Psychology, Neuroscience & Behaviour, McMaster University, Hamilton, Ontario, Canada; University of Chicago, United States of America

## Abstract

Tactile acuity is known to decline with age in adults, possibly as the result of receptor loss, but less is understood about how tactile acuity changes during childhood. Previous research from our laboratory has shown that fingertip size influences tactile spatial acuity in young adults: those with larger fingers tend to have poorer acuity, possibly because mechanoreceptors are more sparsely distributed in larger fingers. We hypothesized that a similar relationship would hold among children. If so, children’s tactile spatial acuity might be expected to worsen as their fingertips grow. However, concomitant CNS maturation might result in more efficient perceptual processing, counteracting the effect of fingertip growth on tactile acuity. To investigate, we conducted a cross-sectional study, testing 116 participants ranging in age from 6 to 16 years on a precision-controlled tactile grating orientation task. We measured each participant's grating orientation threshold on the dominant index finger, along with physical properties of the fingertip: surface area, volume, sweat pore spacing, and temperature. We found that, as in adults, children with larger fingertips (at a given age) had significantly poorer acuity, yet paradoxically acuity did not worsen significantly with age. We propose that finger growth during development results in a gradual decline in innervation density as receptive fields reposition to cover an expanding skin surface. At the same time, central maturation presumably enhances perceptual processing.

## Introduction

In touch, as in other senses, an individual's perceptual acuity is not static throughout life. Among adults, many studies have shown a consistent decline with age in passive tactile spatial acuity, the ability to perceive the fine structure of a stimulus surface pressed against the stationary fingertip [1-5]. This age-associated decline in tactile acuity may result from peripheral mechanoreceptor loss [6,7] and/or changes in central perceptual circuits. When a structured surface contacts the fingertip, it evokes a spatially modulated discharge pattern in the population of underlying slowly adapting type-1 (SA1) mechanoreceptors, a peripheral *neural image* of the stimulus. This neural activity image is transmitted to the CNS, where it is sequentially processed within brainstem and thalamic nuclei, the primary somatosensory cortex, and areas beyond, ultimately resulting in a conscious percept. Clearly, accurate perception depends on both peripheral and central processes, but the fidelity of the initial neural image necessarily sets an upper limit on perceptual accuracy. Thus, the receptor density in a skin region ultimately constrains the spatial acuity achievable with that region, and any decrease in receptor density will tend to result in a decline in acuity. 

The decline in tactile acuity with age has been well characterized among adults, but less is known about how tactile perception develops in childhood. Indeed, the literature is somewhat conflicting even on the basic question of whether tactile acuity improves, declines, or remains unchanged with age early in life [1,8-10]. During childhood, both peripheral (body growth) and central (maturation of perceptual circuits) factors could plausibly cause age-related tactile acuity changes. Two previous studies from our laboratory implicated fingertip size as a predictor of tactile spatial acuity among young adults. We found that index finger tactile spatial acuity improved progressively with diminishing fingertip surface area [11] and that fingertip surface area set a limit on the tactile spatial acuity that could be achieved through training [12]. Together with histological data [6,13-15], these findings supported the hypothesis that cutaneous mechanoreceptors are more closely spaced in smaller fingers. If adults with smaller fingers have better tactile spatial acuity, would the tactile spatial acuity of children be even better than that of adults, and would tactile acuity decline with age as children's fingers grow?

We hypothesized that fingertip growth during development would increase tactile receptor spacing [6,13], with consequent reduction in the fidelity of the peripheral neural image. However, whether tactile spatial acuity would decline with age was unclear, because concomitant CNS maturation might result in more efficient perceptual processing. To investigate, we assessed the tactile spatial acuity of participants aged 6-16 years, and measured each participant's dominant index fingertip surface area, volume, sweat pore spacing, and temperature. Sweat pore spacing was of interest because the Merkel cells innervated by SA1 mechanoreceptors tend to cluster around the bases of the sweat ducts [16,17]. Skin temperature was of interest because it is known to affect vibrotactile perception [18], and might plausibly vary with fingertip size. We found that children's tactile spatial acuity indeed worsened with increasing fingertip size; nevertheless, and intriguingly, tactile spatial acuity did not decline with age. These findings suggest that during childhood, tactile spatial perception is challenged by fingertip growth but simultaneously benefits from CNS maturation.

## Methods

### Ethics statement

All procedures were approved by the McMaster University Research Ethics Board. Because the participants in these experiments were minors, each participant’s parent provided signed informed consent. In addition, each participant provided signed assent.

### Participants

We tested 116 children ranging from 6 to 16 years of age (57 girls, 59 boys). Participants were free of cuts, calluses or scars on their dominant index finger, as well as conditions that might affect their sense of touch, such as diabetes, cognitive impairment, dyslexia, or neurological disorders. We assessed each participant's hand dominance using a modified version of the Edinburgh Handedness Inventory [19]. We eliminated the data of 14 children (7 girls and 7 boys) from analysis due to their poor concentration scores (see Qualification criterion below). The data of another two participants (one 6-year-old boy and one 11-year-old boy) were eliminated as they withdrew from the study prior to completion of their sensory testing. Thus, the data reported here are from 100 participants, 50 boys and 50 girls (Table 1).

**Table 1:** Participants who qualified for the study (n = 100), by age and sex.

	Age										
Sex	6	7	8	9	10	11	12	13	14	15	16
Girls	2	1	5	5	5	5	5	5	5	5	6
Boys	4	2	5	5	5	5	5	5	5	5	5

### Sensory testing

Participants' passive (finger stationary) tactile spatial acuity was estimated by means of the grating orientation task [20-22]. The participant was seated comfortably with the distal pad of the dominant index finger resting over a tunnel in a table through which the tactile stimuli emerged from below. A fully automated tactile stimulator, described in detail in [23], was used to apply the stimuli and record the participant's responses. Briefly, acetyl stimulus pieces (0.5” diameter; milled in-house) with parallel grooves varying from 0.25 to 3.1 mm (in steps of 0.15 mm) were pressed gently onto the participant’s dominant index fingertip (contact force 50 g; contact duration 1 s; onset velocity 4 cm/s). We defined “vertical” gratings as those with their grooves aligned parallel to the long-axis of the finger, and “horizontal” gratings as those with their grooves aligned perpendicular to the long axis of the finger. Small plastic barriers were affixed on either side of the participant’s finger to prevent lateral scanning movement during testing blocks. In addition, a force sensor was placed on the fingernail to monitor upward and downward finger movement throughout testing. A computer-generated voice alerted the participant if any finger movement was detected, and such trials were automatically discarded from analysis. 

Prior to sensory testing, the participant completed 20 practice trials with auditory feedback. The sensory test consisted of 4 blocks of 40 trials each. The average duration of a testing block was 7 minutes. The computer program paused to require the participant to rest for at least 15 seconds after every 20 trials (halfway through each testing block), for at least 1 minute between blocks, and for at least 5 minutes at the halfway point of the experiment (after the second testing block). Sensory testing occurred via either a two-interval forced-choice (2-IFC) procedure (initial 55 participants tested) or a single-interval yes-no procedure (final 61 participants tested). The switch from the 2-IFC protocol to the single-interval protocol was made when it became apparent that young children were struggling to qualify (see Results), and we thought a single-interval protocol with feedback might be simpler for children. In the 2-IFC procedure, participants discriminated the order of two successive grating stimuli of equal groove width but orthogonal orientation (1 s inter-stimulus interval; stimulus order chosen randomly). The participants indicated the perceived stimulus order (vertical grating first or second) by pressing one of two response buttons with the non-dominant hand. No auditory feedback was given during 2-IFC testing. In the single-interval procedure, participants were randomly presented with either a vertical or horizontal grating, and were asked to identify its orientation with a button press using the non-dominant hand. Auditory feedback (one of two computer tones) was provided following each trial in the single-interval procedure, to signal whether the participant had answered correctly or incorrectly. In addition, a visual label was placed on the response button box to identify the vertical and horizontal grating response buttons; no such labels were present for participants tested with the 2-IFC procedure. 

For both the 2-IFC and the single-interval tasks, groove width was adaptively varied using a modified version of the Bayesian adaptive ψ-method [23,24]. Briefly, we modeled the participant’s discriminability, *d-prime*, as a power function of groove width, and the participant’s sigmoidal psychometric function (proportion correct responding as a function of groove width, *x*), Ψ_*a,b,*δ_ (*x*), as a mixture of a cumulative normal curve and a lapse rate term: 


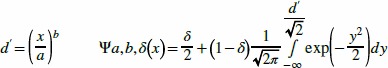


**Figure 1:**
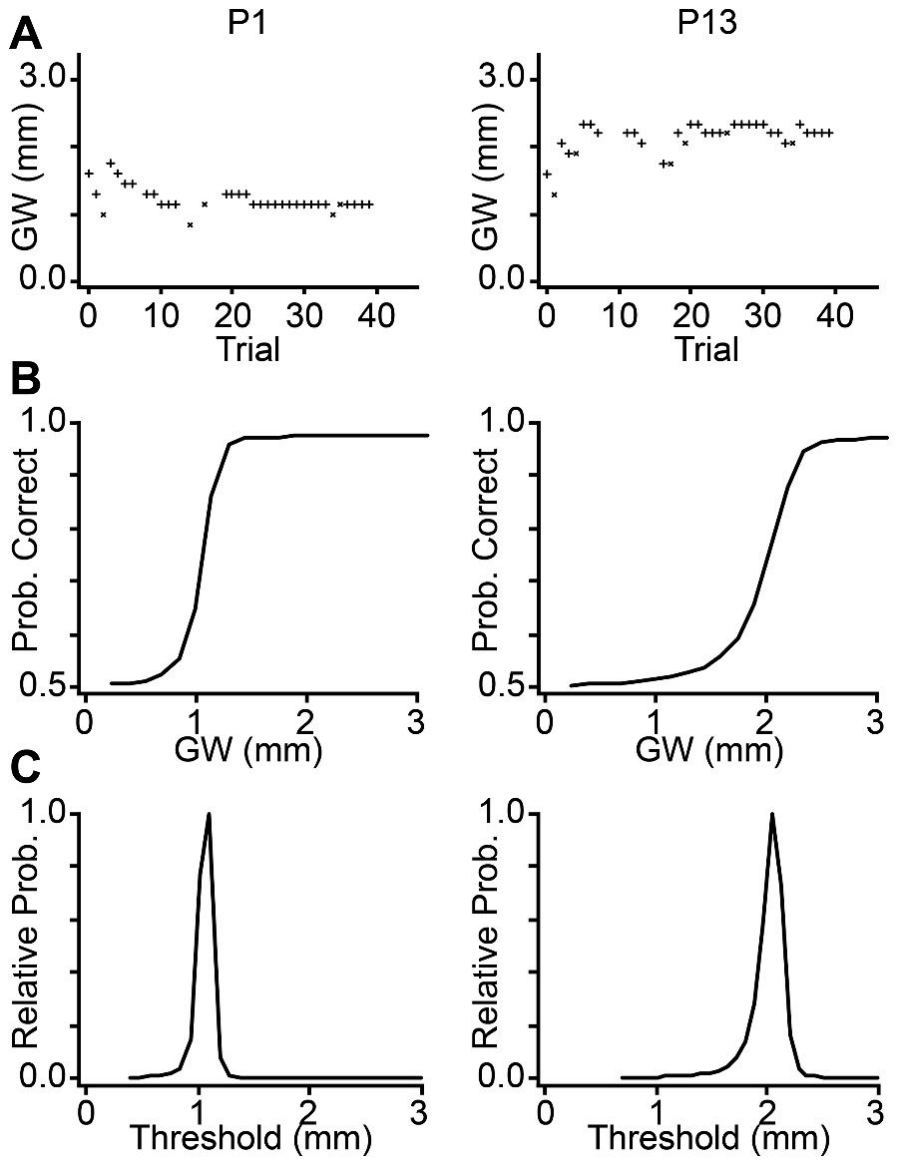
Bayesian adaptive procedure for threshold groove width estimation. Sensory data for two participants (P) are shown in columns: Left panels: P1, female, age 15.9 years, fingertip surface area = 362.8 mm^2^; Right panels: P13, male, age 16.8 years, fingertip surface area = 519.9 mm^2^. (**A**) Each participant's performance plot (+ = correct response, x = incorrect response) on a single testing block. Occasional trials in which the force sensor detected finger movement were automatically discarded (symbols not plotted) so as not to influence the Bayesian adaptive procedure. (**B**) Corresponding best-estimate psychometric functions. (**C**) PDFs over the threshold groove width. Note that, compared to P1, P13 has an upward-shifted performance plot, a rightward shifted psychometric function, and a rightward-shifted threshold PDF, indicative of poorer performance; given the participants' similar ages, this performance difference is likely due to the large difference between the participants' fingertip sizes. GW: groove width; Prob: probability.

The psychometric function is characterized by three unknown shape parameters, which are initially specified by uniform prior probability densities: *a* (position), *b* (slope), and δ (lapse rate). The lapse-rate term accounts for the realistic possibility of occasional attention lapses, resulting in 50% correct response probability, regardless of groove width. The algorithm, which we programmed in LabVIEW for Macintosh (National Instruments) adaptively adjusted groove width from trial to trial, presenting the grating stimulus expected to yield the greatest information regarding the participant’s psychometric function shape parameters (expected entropy minimization). We defined the participant’s GOT threshold as the groove width whose orientation the participant could correctly discriminate with 76% probability. This corresponds to *x* = *a*, at which *d-prime* equals 1 for the 2-IFC task [25], and at which *d-prime* equals 1.35 for the single-interval task. The algorithm returned the best-fitting psychometric function as well as a posterior probability distribution function (PDF) over the *a*-parameter (Figure 1).

For the analysis, we combined the responses from all testing blocks on which the participant was clearly not guessing (see Qualification criterion), and from these combined responses we computed the participant’s joint (*a,b,δ*) posterior PDF. For the 2-IFC task data, we marginalized the joint posterior PDF over the *b* and δ parameters to obtain the participant's *a*-parameter PDF; we took the mean of the *a*-parameter PDF as the participant’s groove width threshold estimate. For the single-interval task, we equivalently derived the groove width at which *d-*prime = 1; this is the 69% correct threshold value for the single-interval procedure [25]. To do this, we marginalized each participant's joint posterior PDF over the δ-parameter, plotted the best-fit psychometric function for each (*a,b*) pair, and interpolated to find the groove width corresponding to 69% correct performance. We then averaged this groove width across the (*a,b*) posterior PDF, and took this as the participant's threshold estimate.

### Qualification criterion

Consistent performance on a psychophysical task demands sustained concentration. We screened participants for concentration by assessing the probability that their performance could have resulted from guessing on each trial, relative to the probability that it could be described by a cumulative normal psychometric function. We call the ratio of these two probabilities the Guessing Bayes Factor (GBF), which we compute as:


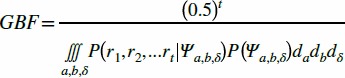


where *r*_*i*_ refers to the participant’s response (correct or incorrect) on the i^th^ trial, *t* is the total number of non-discarded trials in the testing block, and P(Ψ_*a,b,*δ_) is the prior probability density over the psychometric function characterized by parameters *a*, *b*, and δ.

We chose a criterion value of GBF = 0.5 as the cut-off above which we considered a participant not to be concentrating during a given testing block. Thus, only if we were at least twice as confident that a participant was concentrating than randomly pressing buttons did we accept the participant's data from that testing block for further analysis. We applied this criterion on a block-by-block basis. We performed a sensitivity analysis to investigate the effect of different choices of qualification criterion on the statistical results (Table 2). 

**Table 2:** GBF criterion value sensitivity analysis.

	**GBF Criterion**			
**Current study**	1 (n=103)	0.5 (n=100)	0.1 (n=97)	0.01 (n=93)
Fingertip volume, age	p = 0.025, 0.264	p = 0.016, 0.353	p = 0.033, 0.496	p = 0.094, 0.947
	β = 0.277, -0.156	β = 0.304, -0.131	β = 0.263, -0.097	β = 0.196, -0.010
	R^2^= 0.040	R^2^= 0.053	R^2^= 0.043	R^2^= 0.036
Between-ridge pore spacing, age	p = 0.049, 0.541	p = 0.055, 0.818	p = 0.110, 0.983	p = 0.123, 0.701
	β = 0.200, -0.074	β = 0.197, -0.028	β = 0.152, 0.003	β = 0.146, 0.048
	R^2^= 0.029	R^2^= 0.033	R^2^= 0.024	R^2^= 0.031
Fingertip surface area, age	p = 0.059, 0.362	p = 0.031, 0.405	p = 0.073, 0.610	p = 0.129, 1.000
	β = 0.236, -0.137	β = 0.281, -0.124	β = 0.223, -0.078	β = 0.175, 0.000
	R^2^= 0.026	R^2^= 0.042	R^2^= 0.030	R^2^= 0.031
**Including young adults**	1 (n=200)	0.5 (n=197)	0.1 (n=194)	0.01 (n=190)
Fingertip surface area, age	p < 0.001, 0.008	p < 0.001, 0.014	p < 0.001, 0.028	p < 0.001, 0.035
	β = 0.373, -0.265	β = 0.397, -0.245	β = 0.380, -0.219	β = 0.364, -0.210
	R^2^= 0.067	R^2^= 0.079	R^2^= 0.074	R^2^= 0.069

Multiple regression results between independent variables (left column) and log tactile thresholds (dependent variable) for several GBF criterion values (number of participants meeting criterion: n). P-values and beta weights (standardized regression coefficients) are reported in order 'fingertip metric, age'. R^2^: proportion of explained variance. “Including young adults”: data from the present study aggregated with those of Peters et al. [11].

The GBF typically reached very low values, indicative of good concentration among our participants. Furthermore, the GBF did not increase as a function of block number; thus, we are confident that the participants did not progressively lose concentration during the experiment. The median GBF values (all participants who completed the experiment) for testing blocks 1-4 were 0.0041, 0.0018, 0.0018, and 0.0017, respectively.

### Physical skin measurements

We measured the surface area, volume, temperature, and sweat pore spacing of the dominant index fingertip of each participant.

To determine fingertip surface area, we scanned the distal portion of the participant’s dominant index finger with a flatbed scanner (Epson Perfection 1260). This scanning procedure is identical to that used by Peters et al 2009. The participant placed their hand on a glass scanning surface in prone position, and the distal finger pad, from the tip of finger to the distal inter-phalangeal crease, was optically imaged at 400 dpi. Fingertip surface area was digitally measured from these images using ImageJ v10.2 (National Institutes of Health). Fingertip surface area was measured by two naive observer (PS and SP); we report the average of the observers' measurements.

To measure index finger volume, we determined how much water the fingertip displaces when submerged up to the distal inter-phalangeal crease in a plastic 20 mL graduated cylinder. The cylinder was filled to the top with room-temperature water; insertion of the finger caused a volume of water to spill out that was equal to the volume of the fingertip. We then used a USB microscope with a polarized 30X lens (ProScope HR; Bodelin Technologies) to image the waterline after the finger was withdrawn. These measurements were made digitally using GraphClick v3.0 to define the graduated cylinder tick marks above and below the water line and to measure the water line's linear position (at its lowest point) between those bracketing tick marks. To improve visibility of the water line in the ProScope images, red food colouring was added to the water and a blank piece of white paper was held against the side of the graduated cylinder opposite to the ProScope lens. This measurement was repeated 4 times and the resulting fingertip volume measurements were averaged together for use in the analysis.

To measure skin surface temperature, we used a thermistor (ON-408-PP, Omega Engineering, USA). These temperature-dependent resistors are designed for accurate skin surface temperature measurement within +/- 0.1 °C. We made three separate temperature measurements: once before sensory testing began, once at the halfway point (after completion of testing block 2), and once upon completion of the sensory testing; these three measurements were averaged together for use in the analysis.

To measure sweat pore density, we coated participants' dominant index fingertip with water-based paint (Crayola Water Colours) and optically imaged the distal pad at 2400 dpi with a flatbed scanner (EPSON Perfection, 1260). We measured center-to-center sweat pore spacing from these scans using ImageJ. Because we previously found that sweat pore spacing between adjacent fingerprint ridges differed from sweat pore spacing within individual ridges [11], we estimated average between-ridge (μ_b_) and within-ridge (μ_w_) sweat pore spacing separately, from 20 measurements of each. Two observers performed these measurements, an author (RMP) and a naive observer (AB), and we averaged their measurements. We estimated sweat pore density, ρ (pores/mm^2^), as:

$$\rho =\frac{1}{\mu_{b}\mu_{w}}$$

### Statistical analyses

All statistical analyses were conducted using SPSS version 20 for Mac (IBM Corporation) with an alpha level of 0.05. For ANCOVA, we used type III sum of squares. Reported p-values are two-tailed unless otherwise stated. For analyses of the effect of age on fingertip size metrics, and of fingertip size metrics on tactile threshold, we used one-tailed p-values, because we had directional alternative hypotheses. Specifically, we predicted that fingertips would grow with age, and that tactile thresholds would increase with fingertip size. For other analyses, including the effect of age on skin temperature and on tactile thresholds, we used two-tailed p-values, because we had no strong prediction regarding the direction of these effects. 

We log transformed participants’ tactile thresholds prior to analysis, because the measured thresholds, as well as the standardized residuals from linear regressions with measured threshold as the dependent variable, were non-normally distributed as indicated by the Kolmogorov-Smirnov (KS) test [tactile thresholds (p < 0.001); residuals from linear regressions between thresholds and fingertip surface area (p < 0.001), volume (p = 0.004), temperature (p = 0.002), between-ridge sweat pore spacing (p = 0.001), within-ridge sweat pore spacing (p = 0.001), sweat pore density (p < 0.001), age (p = 0.001)]. Log-transformation greatly improved normality, with KS tests revealing no significant violations of normality [log thresholds (p = 0.07); residuals from linear regressions between log thresholds and fingertip surface area (p = 0.096), volume (p = 0.2), temperature (p = 0.085), between-ridge sweat pore spacing (p = 0.2), within-ridge sweat pore spacing (p = 0.124), sweat pore density (p = 0.065), age (p = 0.181)]. In one analysis (see Results), we aggregated the data from the current study with those from Peters et al. [11]; for that purpose, we first log-transformed the thresholds reported in [11], which improved the normality of the residuals for those data as well. 

We performed multiple linear regressions on log tactile thresholds with age and physical fingertip metrics as predictor variables. Because these fingertip metrics themselves correlated with age (see Results), we calculated variance inflation factors (VIF) [26] to assess whether collinearity was not problematically high. All VIF were less than 2.3, indicating that the degree of collinearity among independent variables was well within acceptable limits [26].

## Results

### Participant concentration and task difficulty

**Figure 2:**
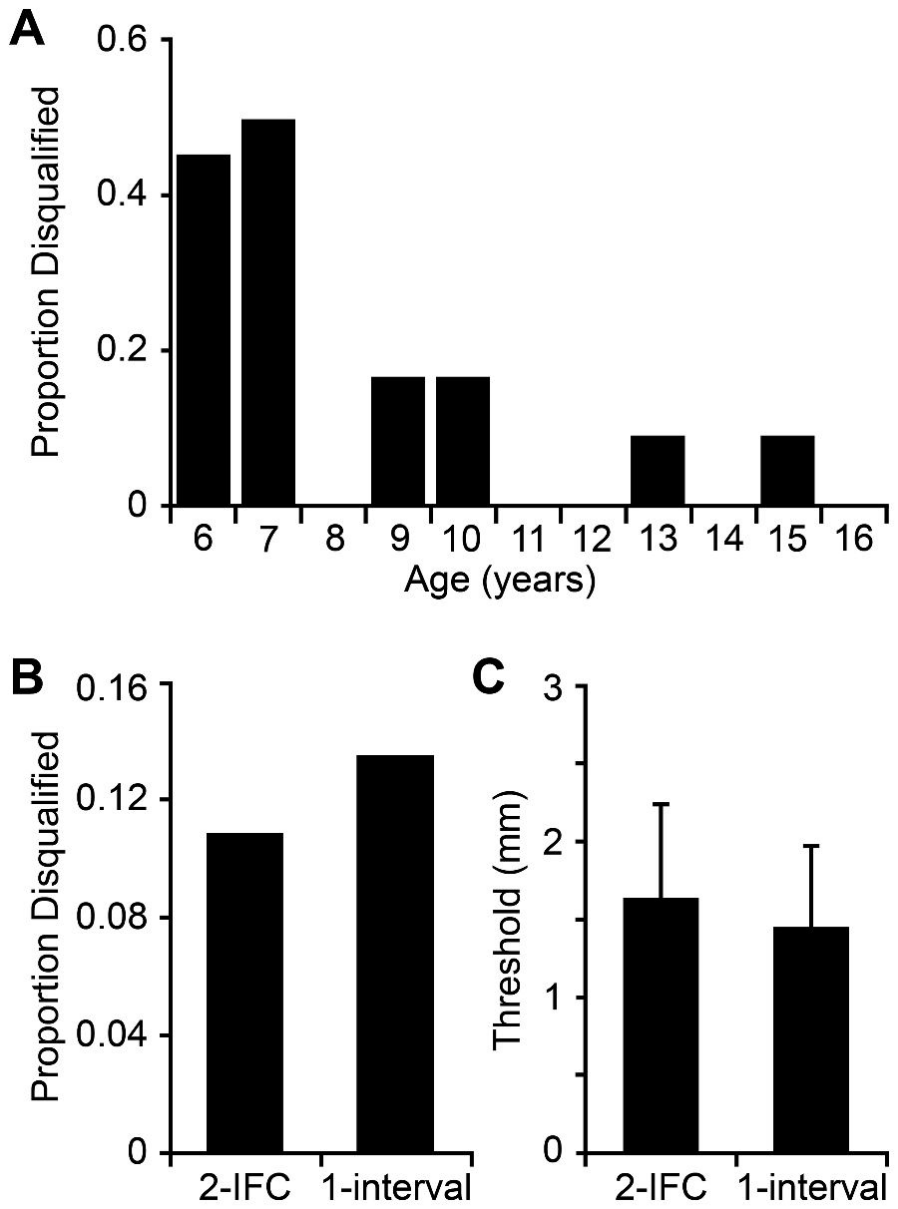
GBF disqualification analysis and comparison between testing protocols. (**A**) Proportion of participants in each age bracket for whom all four end-of-block GBFs exceeded the criterion value of 0.5. (**B**) Proportion of participants disqualified using the two testing protocols. (**C**) Average thresholds of qualifying participants on the two testing protocols (error bars: 1 SD).

We found that the youngest participants were much more likely to struggle with the sensory testing. A chi-squared test revealed that the proportion of participants eliminated due to poor concentration (see Methods) was significantly greater than zero among 6 year olds (X^2^ = 6.471, p = 0.011) and 7 year olds (X^2^ = 4.000, p = 0.046) (Figure 2A). Although we had hoped that the single-interval stimulus procedure would prove easier for the younger participants, a chi-squared test revealed that the proportion of participants who failed to qualify on the 2-IFC task did not differ significantly from the proportion who failed to qualify on the conceptually simpler single-interval task (X^2^ = 0.186, p = 0.667) (Figure 2B). Furthermore, among qualifying participants, the mean tactile threshold on the 2-IFC experiments (1.63 mm; SD = 0.60 mm) did not differ significantly from that on the single-interval experiments (1.45 mm, SD = 0.52 mm) (Figure 2C). An ANCOVA with testing protocol and sex as between subject factors, age as a covariate, and log threshold as the dependent variable revealed no significant effects of any factor (testing protocol, p = 0.117; age, p = 0.544; sex, p = 0.462). Therefore, for subsequent analyses we used the aggregate data from the two protocols.

### Fingertip growth during development

**Figure 3:**
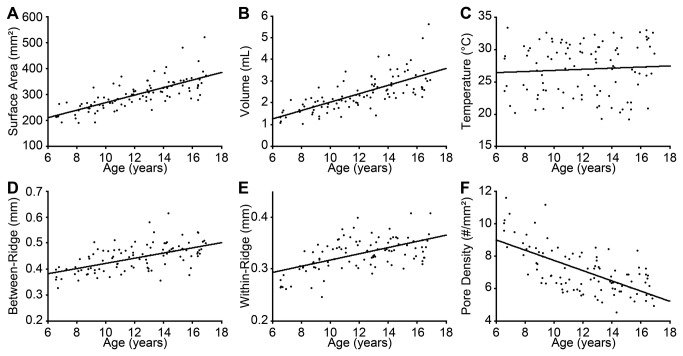
Fingertip growth with age. Six different fingertip metrics are plotted against age. (**A**) surface area, (**B**) volume, (**C**) temperature, (**D**) between-ridge sweat pore spacing, (**E**) within-ridge sweat pore spacing, (**F**) sweat pore density. Black lines: least-squared linear fits.

To characterize the physical changes in the fingertip that occur during development, we conducted separate linear regressions between participant age and the six fingertip metrics collected in this study. These revealed significant positive relationships between participant age and fingertip surface area (r = 0.744, one-tailed p < 0.001; slope 14.560 mm^2^/year), volume (r = 0.709, one-tailed p < 0.001; slope 0.195 mL/year), between-ridge sweat pore spacing (r = 0.572, one-tailed p < 0.001; slope 0.010 mm/year), and within-ridge sweat pore spacing (r = 0.555, one-tailed p < 0.001; slope 0.006 mm/year). There was a significant negative relationship between age and estimated sweat pore density (r = -0.652, one-tailed p < 0.001; slope -0.316 pores/mm^2^/year). Thus, fingertips enlarged and sweat pore spacing increased with age. Fingertip temperature did not correlate significantly with age (p = 0.529) or with fingertip surface area (p = 0.145), volume (p = 0.093), or surface-to-volume ratio (p = 0.158) (Figure 3).

### The effects of age and fingertip characteristics on tactile spatial acuity

**Figure 4:**
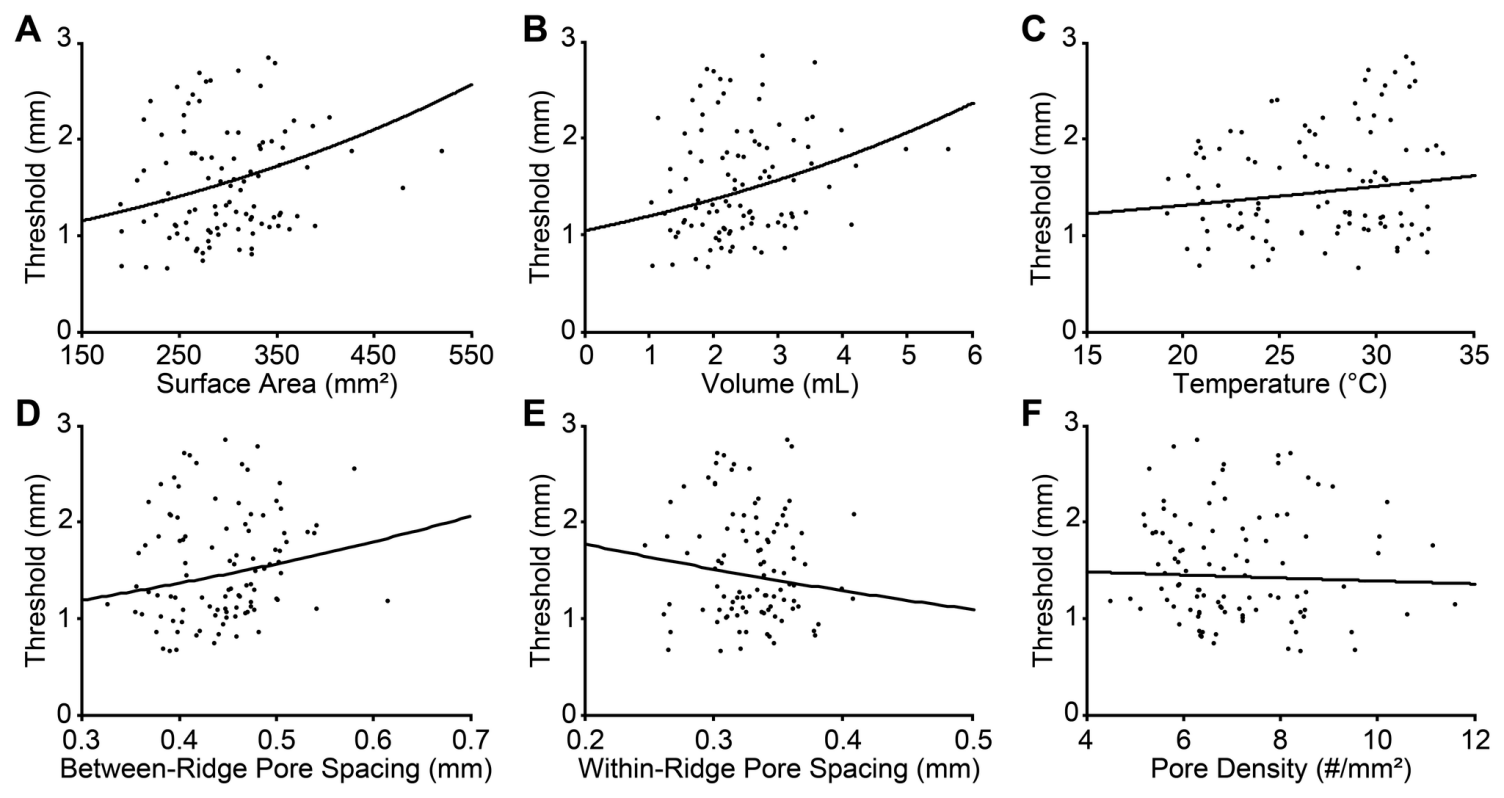
Tactile spatial acuity dependence on fingertip metrics. (**A**) surface area, (**B**) volume, (**C**) temperature, (**D**) between-ridge sweat pore spacing, (**E**) within-ridge sweat pore spacing, (**F**) sweat pore density. Black curves: best-fit exponential functions from multiple regressions relating log threshold (dependent variable) to fingertip metric and age (independent variables).

Next, we addressed the primary questions of this study: do age and/or fingertip characteristics significantly influence tactile spatial acuity among children? To investigate the effect of age, we first conducted a simple linear regression between age and log tactile threshold; this showed no significant effect of age on tactile spatial acuity among our participant sample (p = 0.403). We next conducted separate multiple linear regressions using each of the six physical fingertip metrics together with age as independent variables, and log tactile threshold as the dependent variable. These analyses revealed significant effects of fingertip surface area (r = 0.206, one-tailed p = 0.031) and volume (r = 0.230, one-tailed p = 0.016), and a marginally significant effect of between-ridge sweat pore spacing (r = 0.182, one-tailed p = 0.055) (Figure 4). Age did not significantly predict tactile acuity in these analyses (p > 0.3 in all cases), although interestingly the beta weights for age were consistently negative, suggesting a non-significant trend for acuity to improve a function of age (see Table 2). Thus, among participants 6 to 16 years old, greater fingertip size was associated with significantly poorer tactile spatial acuity, whereas the effect of age was not significant.

### Aggregate analysis with the data of Peters et al. (2009)

**Figure 5:**
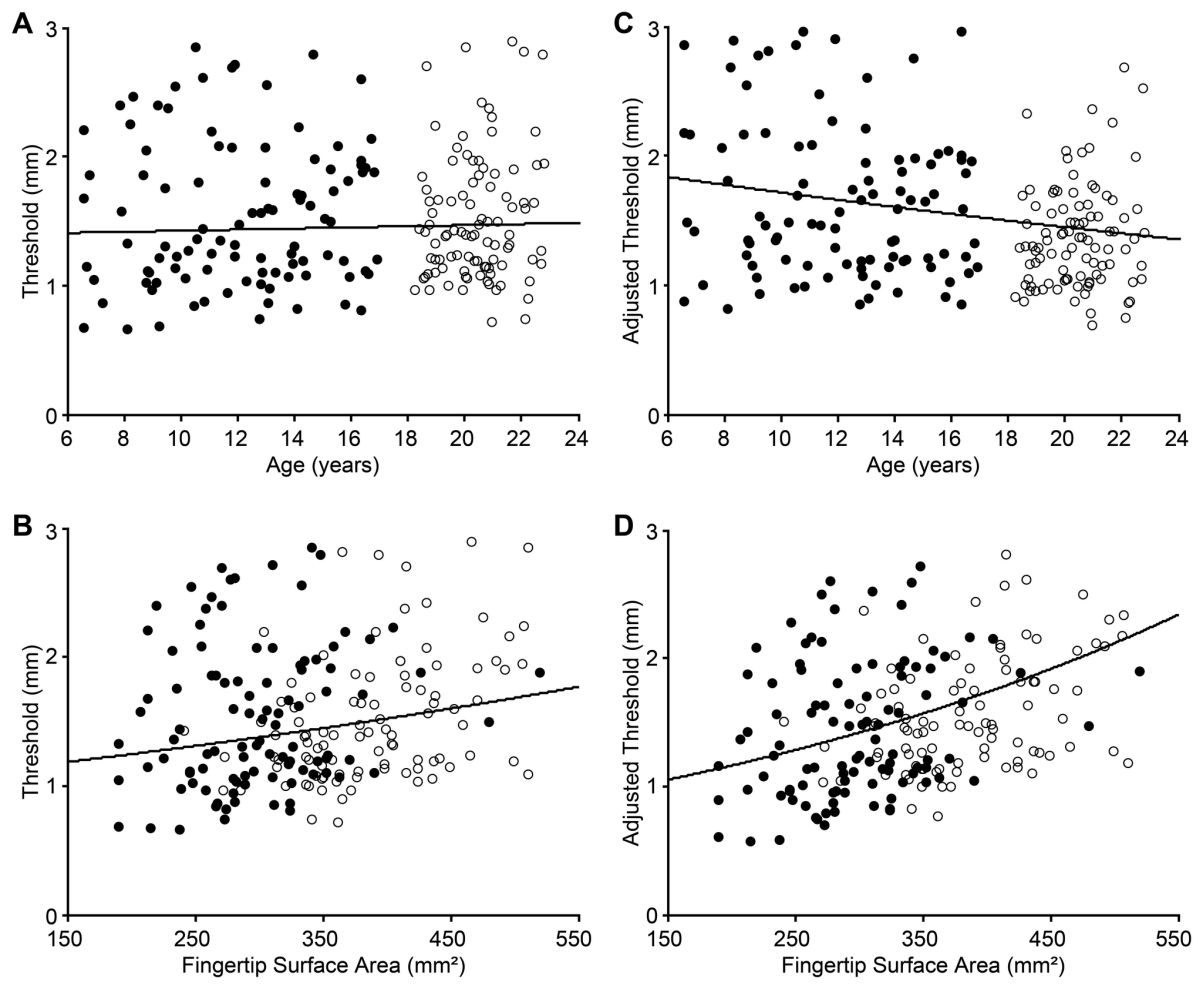
Tactile spatial acuity from childhood into adulthood. Data from the current study (filled circles) are plotted together with those from Peters et al. [11] (open circles). (**A**) and (**B**) show simple linear regressions. (**A**) threshold vs. age. (**B**) threshold vs. fingertip surface area. (**C**) and (**D**) show results of a multiple regression with both age and surface area as independent variables. (**C**) surface area-adjusted threshold vs. age. (**D**) age-adjusted threshold vs. surface area. In (**C**) and (**D**), thresholds were respectively adjusted to the mean surface area and mean age of the aggregate participant sample. Black solid curves in all panels: least-squared exponential fits. In (**A**) and (**C**), for plotting purposes only, we have omitted the data from the oldest participant, a 27.29 year-old from [11] (threshold 1.22 mm, area-adjusted threshold, 1.30 mm).

To further investigate whether tactile spatial acuity changes with age from childhood into early adulthood, we aggregated the data from the 100 qualifying children in the present study with those of 97 young adults (ages 18 - 27 years) whom we had tested in a previous 2IFC GOT study using the same automated testing apparatus and tactile stimulus pieces [11] (Figure 5). When considered alone, age again failed to predict tactile spatial acuity (Figure 5A). A univariate linear regression revealed no significant effect of age on log tactile thresholds in the aggregated dataset (p = 0.590). The results were distinct, however, when we considered age along with fingertip surface area (the sole fingertip size metric recorded for all participants by Peters et al. [11]). A multiple regression on the aggregated log thresholds revealed significant effects of both age (*t* = -2.490, p = 0.014) and fingertip area (*t* = 4.042, one-tailed p < 0.001), with opposite directionality (Figure 5C, D). Tactile spatial acuity improved significantly with age (rate = 0.017 log mm threshold decrease/year; β = -0.245) and worsened significantly with increasing fingertip area (rate = 0.002 log mm threshold increase/mm^2^ surface area; β = 0.397). These findings are consistent with the intriguing hypothesis that two concomitant effects are at play during development: a progressive worsening of acuity as fingertips grow, and a progressive improvement in acuity as the CNS becomes more efficient at tactile processing; together, these factors tend to cancel the effect of age – considered alone – on tactile spatial acuity during development. 

## Discussion

In this cross-sectional study, we found that tactile spatial acuity among children worsens with increasing fingertip size, as reported previously in young adults [11]. Additionally, by combining the data from the present and a previous study, we discovered that fingertip size and age exert opposite effects on tactile acuity when both variables are considered together. Statistically, at a given age, acuity worsens with increasing fingertip size; at a given fingertip size, acuity improves with increasing age. These findings suggest that two factors act concomitantly during development to influence tactile spatial acuity: fingertip growth results in a gradual decline in mechanoreceptor density, and CNS maturation results in more efficient sensory processing. 

### Technical considerations in testing young children

Some methodological observations from our experiences testing young children may prove useful to other researchers who are considering psychophysical studies with young participants.

A concern in testing young children is that the psychophysical task may prove overly cognitively demanding [27]. After testing 55 participants using a 2-IFC procedure, we modified our protocol in an effort to make the experiment as easy as possible to understand and perform. We tested another 61 participants using a single-interval stimulus procedure, providing auditory feedback on every trial, and identifying the response buttons with visual vertical and horizontal gratings. Despite these modifications, we found no significant differences in tactile acuity as measured on the two tasks. In light of this equivalence in performance, and because the 2-IFC procedure is robust against criterion effects [25], we recommend that researchers use the 2-IFC procedure in future GOT studies with children, as with adults.

Another concern in testing young children is that the data may become corrupted with spuriously high threshold measurements produced by children who are simply not concentrating on the task. Sensory investigators have recognized the potential hazards associated with the limited attention spans of young children [27]. However, the degree of this problem has been difficult to assess, because psychophysical procedures do not typically provide an objective concentration measure. We overcame this limitation by measuring the guessing Bayes factor on each testing block, thereby detecting participants who were unable to concentrate consistently. We found that only the 6 and 7 year-old groups significantly exceeded GBF criterion. Based on this observation, we recommend against testing such young children on the GOT and similarly demanding tactile tasks, unless the GBF is also measured. 

Our interpretation of the GBF relies only on the assumption that a participant, if concentrating, would be able to perform our task non-randomly. A hypothetical participant who was unable to distinguish even the largest stimulator in our collection (groove width = 3.1 mm) would respond as if guessing on every trial. We consider it unlikely, however, that the children whose performance crossed our GBF criterion were in this category. Our reasoning, though anecdotal, is compelling. With experience, the experimenter was able to predict, even before the experiment began, which children would fail the GBF criterion. Typically these children were highly energetic upon their entrance to the lab ("bouncing off the walls"), were easily distracted, and ignored simple instructions (e.g., “please hold your finger still”). They often shifted about in the experiment chair, appeared bored, and failed to ask clarifying questions. In short, they showed independent signs of poor concentration. This stands in stark contrast to the generally calm and engaged demeanor of the children who passed the GBF criterion.

### Effect of fingertip size on tactile spatial acuity in childhood

We found that fingertip size is a significant predictor of tactile spatial acuity in childhood, as shown previously in young adulthood [11]. This result is consistent with the hypothesis that cutaneous mechanoreceptors become more widely spaced as the finger grows. This change in spacing would maintain sensory coverage throughout the surface of the fingertip. However, the consequent reduction in receptive field density and probable increase in receptive field size would cause a decline in tactile spatial acuity. In addition, it is conceivable that receptor depth increases with finger growth. Receptors deeper beneath the skin surface would experience less strain from a tactile stimulus [28,29], with consequent reduction in the quality of the peripheral neural image, leading to diminished acuity.

SA1 afferents innervating Merkel cell mechanoreceptors convey the fine spatial information that underlies performance on passive tactile spatial tasks such as the GOT [30,31]. Therefore, the most probable neural explanation for the decline in tactile acuity with increasing fingertip size is that the Merkel cells become more widely spaced as fingers grow. To our knowledge, no anatomical evidence currently exists regarding the change in density of Merkel cells in humans with age. However, several studies have reported that the relatively easily visualizable Meissner's corpuscles, which mediate low-frequency vibration perception [30], are more sparsely distributed in larger fingers [13-15]. In a cross-sectional anatomical study, Bolton et al. [13] further showed that the density of Meissner's corpuscles, measured in the little finger, declined with age from childhood through adulthood. Bolton et al. [13] proposed that the decline in Meissner density during childhood is due to finger growth; they note that the continuing (yet slower) decline during adulthood is of unclear cause.

We found that between-ridge sweat pore spacing was a marginally significant predictor of acuity, whereas within-ridge sweat pore spacing was not. Between-ridge sweat pore spacing may be more tightly linked to average afferent receptor spacing and receptive field size. Pare et al. [32] showed that in the distal pads of non-human primates, about 80% of Merkel cells form clusters of 30 - 70 μm in diameter that stud the basal layer of intermediate ridges; the remaining 20% of Merkel cells do not cluster together but rather form chain-like arrangements that are 300 - 500 μm in length. Aβ afferents can branch to up to three adjacent intermediate ridges [33]. However, within each intermediate ridge, Aβ afferents can branch to a Merkel cell cluster surrounding the adjacent sweat duct or to a cluster or chain-like Merkel cell arrangement between adjacent sweat ducts [17,32]. Thus, the diversity of innervation targets within an intermediate ridge likely renders our within-ridge sweat pore spacing a poorer proxy than our between-ridge sweat pore spacing for receptive field spacing and size, and therefore, a poorer predictor of tactile spatial acuity.

The effects we have observed of fingertip size on tactile spatial acuity, while significant, are weaker than those observed previously by our laboratory among young adult participants [11]. Clearly, fingertip size is not the sole determinant of tactile spatial acuity during childhood; central factors must also play a role.

### Effect of age on tactile spatial acuity in childhood and adulthood

Age did not significantly affect tactile spatial acuity among the children tested in the present study, nor did tactile acuity correlate with age alone when the data from the present study were aggregated with those from the young adults tested in Peters et al. [11]. However, we uncovered a beneficial effect of age on tactile spatial acuity when we analyzed the aggregated data set with a multiple regression that included age along with finger size. Our findings suggest that, as fingertips grow during childhood, afferent receptor density declines, diminishing the fidelity of the peripheral neural image that is transmitted into the CNS for perceptual processing; at the same time, however, as CNS pathways and circuits mature, central perceptual processing likely improves with age. Because of these opposing effects, the influence of age, considered alone, is weak. The beneficial effect of age on tactile spatial acuity becomes apparent once fingertip size is controlled. 

To our knowledge, only two other research groups have investigated age-related tactile spatial acuity change in children. Using a grating orientation task, Bleyenheuft et al. [8] reported that tactile spatial acuity improved with age, specifically 10 to 16 year old participants outperformed 6 to 9 year olds. Similarly, Bleyenheuft et al. [9] found that acuity improved from ages 4 to 17 years. Using a gap-detection task, Stevens and Choo [1] reported that tactile spatial acuity worsened with age, specifically 8 to 14 year old participants outperformed young adults (18 to 28 years old). Because of the different age ranges considered, these studies are not necessarily in disagreement; rather, taken together, the studies suggest a non-monotonic effect of age on tactile acuity, with acuity initially improving and then worsening with increasing age. Indeed, previous research from our laboratory and others shows that during adulthood tactile spatial acuity consistently worsens with age [1-5], perhaps because of progressive loss of mechanoreceptors [6,7].

### Possible implications for vibrotactile perception

Although our study concerned tactile spatial acuity, we can speculate about the relevance of our findings to the performance of children on non-spatial tactile tasks. Specifically, our results may help to interpret data on fingertip vibrotactile sensitivity in children compared to adults. Bernstein et al. [34] reported that young adults outperformed children at detecting low-frequency (e.g., 10 Hz and 20 Hz) vibrations, which activate rapidly adapting type I (RA1) receptors, whereas the two groups performed equivalently at detecting higher frequency vibrations, which activate Pacinian (PC) receptors. Similarly, Güçlü and Öztek [10] found no significant differences between the detection thresholds of children and young adults when the PC channel was activated by 40 Hz or 250 Hz vibration. In addition, by masking the PC channel, they were able to test participants’ ability to detect 40 Hz vibrations with the RA1 channel. Here, they found a marginally significant trend (p = 0.053) for adults to outperform children. Taken together, these studies suggest that the superior fingertip vibrotactile sensitivity of adults is specific to the RA1 channel; when the PC channel is activated, children perform equivalently to adults. 

Why does vibrotactile sensitivity improve from childhood to adulthood for stimuli that activate the RA1 channel but not for stimuli that activate the PC channel? Our findings suggest a plausible if speculative explanation. We propose that, just as for SA1 receptors, smaller fingers have a higher density of RA1 and PC receptors. Consequently, a contactor of given surface area will overlie more receptors in the fingers of children than of adults. Unlike the RA1 channel, the PC channel is known to exhibit pronounced spatial summation, such that detection thresholds decrease when the stimulus activates a greater number of PC receptors [35]. Therefore, just as occurs in spatial perception, the detection of vibratory stimuli with the PC channel will benefit from age-related central maturation but simultaneously suffer from fingertip growth that reduces PC receptor density. A plausible result of these dual opposing processes is that sensitivity to vibrations that activate the PC channel remains approximately constant over age. Fingertip growth would similarly reduce the density of RA1 receptors, but this might not markedly impair the detection of vibrations that activate the RA1 channel, in which spatial summation is reportedly minimal or absent [36]; in detecting such vibrations, adults would therefore tend to outperform children. 

### Comparison to other sensory systems

We have concluded that during childhood, growth in the somatosensory receptor sheet and central neural maturation exert opposing influences on tactile acuity. Do analogous phenomena occur in other sensory systems? It is surely reasonable to consider that central maturation during childhood improves perceptual processing in audition and vision, as well as in touch. For instance, post-mortem neurofilament staining revealed that the axonal density in superficial layers of human auditory cortex does not reach adult levels until 11-12 years of age [37]. More recently, diffusion tensor imaging (DTI) showed evidence of widespread maturation in forebrain white matter and deep gray matter in childhood and adolescence; areas of ongoing maturation included the thalamus, the posterior limb of the internal capsule (containing somatosensory thalamocortical axons), and frontal association fibers [38]. A longitudinal DTI study revealed development of frontal tracts associated with cognitive processing extending into participants’ twenties [39]. These anatomical findings likely underestimate the extent of ongoing maturation, as many changes would go undetected by DTI, such as subtle experience-dependent local circuit refinement. Thus, we suspect that visual, auditory, and somatosensory perceptual processing and associated decision circuits benefit from ongoing central neural maturation during childhood. 

In the case of vision and audition, however, anatomical evidence suggests that the beneficial perceptual effects of central maturation are not countered by concomitant changes in the size of the receptor sheet. The auditory receptor sheet, the basilar membrane, has adult length at birth [40], and though more research is needed, the inner and outer hair cells and their innervation also appear adult-like at birth [41]. The visual receptor sheet, the retina, does expand postnatally, as it is stretched by the growth of the eye [42,43]. Nevertheless, the resulting increase in retinal surface area is unlikely to adversely affect central visual acuity throughout childhood. By age 6 years, retinal surface area is already approximately 900 mm^2^ [42], i.e., 90% of its adult value [44]. The modest further increase in retinal surface area from 6 years to adulthood contrasts to the growth of the fingertip, where we find that surface area approximately doubles, from around 200 mm^2^ at 6 years to around 400 mm^2^ in adulthood (Figures 3 and 5). Furthermore, retinal stretching is thought to occur primarily in the peripheral retina rather than in the fovea [43]. Most importantly, as a result of cone migration, foveal photoreceptor density actually *increases* from birth into early childhood, approaching adult levels at some point after 4 years of age [42,45,46]. 

Therefore, in audition and vision, unlike in touch, we do not expect growth of the receptor sheet during childhood to progressively degrade the peripheral neural image. Rather, we expect central maturation, unopposed and perhaps even assisted by peripheral changes, to improve sensory acuity. Consistent with this notion, studies have shown that adults clearly outperform children on spatial visual tasks [47,48] and on auditory tasks such as frequency and duration discrimination [49,50].

## Conclusion

Our results support the hypothesis that two opposing influences act on tactile spatial perception during childhood: fingertip growth diminishes the fidelity of the peripheral neural image, but CNS maturation enhances perceptual processing. We note that the perceptual data show large individual variability (Figures 4 and 5), and indeed much variance remains unexplained (see R-squared values in Table 2). Future research will continue the important search for both peripheral and central sources of individual variability in tactile perception. We note that peripheral factors in addition to those associated with finger size may play an important role in tactile spatial acuity. Among plausible candidates, skin moisture deserves further attention [18,51]. Meanwhile, given the results of the present study, we recommend that not only age but also fingertip size be taken into account when tactile spatial acuity is compared across individuals.
